# Cutaneous leukemic infiltration following varicella - a case of Wolf's isotopic response*

**DOI:** 10.1590/abd1806-4841.20164686

**Published:** 2016

**Authors:** Ana Brasileiro, André Lencastre, Alexandre João, Ana Fidalgo

**Affiliations:** 1 Hospital S. António Capuchos - Centro Hospitalar Lisboa Central – Lisbon, Portugal

**Keywords:** Chickenpox, Leukemic infiltration, Leukemia, lymphocytic, chronic, B-cell

## Abstract

Wolf's isotopic response designates the appearance of two subsequent unrelated dermatoses in the same anatomic location. We report the case of a 51-year-old man with a medical history of chronic lymphocytic leukemia without known extra-hematopoietic involvement. The patient developed a disseminated papulo-vesiculous eruption, diagnosed as varicella. Few days after recovering, an erythematous and violaceous papular dermatosis with histopathological examination compatible with leukemic infiltration appeared on the scars of previous herpetic lesions. Complete remission was obtained under systemic corticotherapy, without cutaneous recurrence or blastic transformation. Wolf's isotopic response is attributed to a localized immunologic imbalance following a certain stimulus. In this patient, herpetic infection acted as a local spur for inaugural cutaneous leukemic infiltration, with no impact on the prognosis for the underlying disease.

## INTRODUCTION

Wolf's isotopic response designates the appearance of a new dermatosis at the site of an already healed unrelated skin disease.^1^ The initial dermatosis is usually a herpetic infection, in particular herpes zoster.^2^ It acts as a presumed trigger for local immune dysregulation – excessive or defective immune responses – implicated in the subsequent dermatosis. The most common isotopic responses – which appear days or years after the initial disease has healed – are granulomatous reactions, malignant infiltrates, lichenoid dermatosis or infections.^1,3^

## CASE REPORT

**Figure 1:**
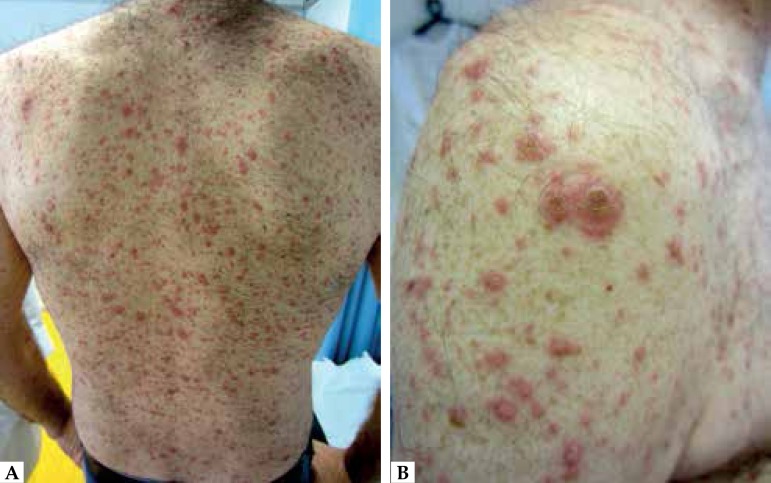
**A.** Widespread papulo-vesiculous eruption; **B.** Grouped confluent vesicles and bullae

A 51-year-old man diagnosed with B-cell chronic lymphocytic leukemia (B-CLL) without extra hematopoietic infiltration reported to the clinic. The patient had undergone several chemotherapy cycles – including rituximab, cyclophosphamide, fludarabine and alemtuzumab – without achieving complete clinical remission. Three years after the diagnosis, while on cyclophosphamide and prednisolone, the patient developed a sudden widespread non-pruritic papulo-vesiculous dermatosis. Histopathological examination revealed intraepidermal vesicles with a mixed inflammatory infiltrate and solitary keratinocytes within the blisters (Figure 1). We also observed multinucleated giant cells with viral nuclear inclusions and margination of chromatin, diagnosing herpetic infection.

Laboratory results, namely negative IgG anti-varicella-zoster virus, suggested varicella instead of disseminated zoster infection. The lesions healed under intravenous acyclovir 30 mg/kg/day for 8 days.

**Figure 2:**
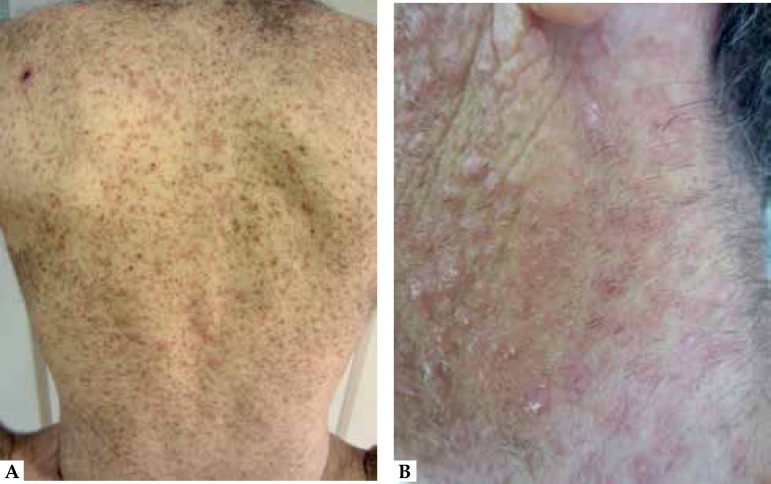
**A.** Widespread erythemato-violaceus papular dermatosis; **B.** Multiple monomorphic *papules de novo*

**Figure 3:**
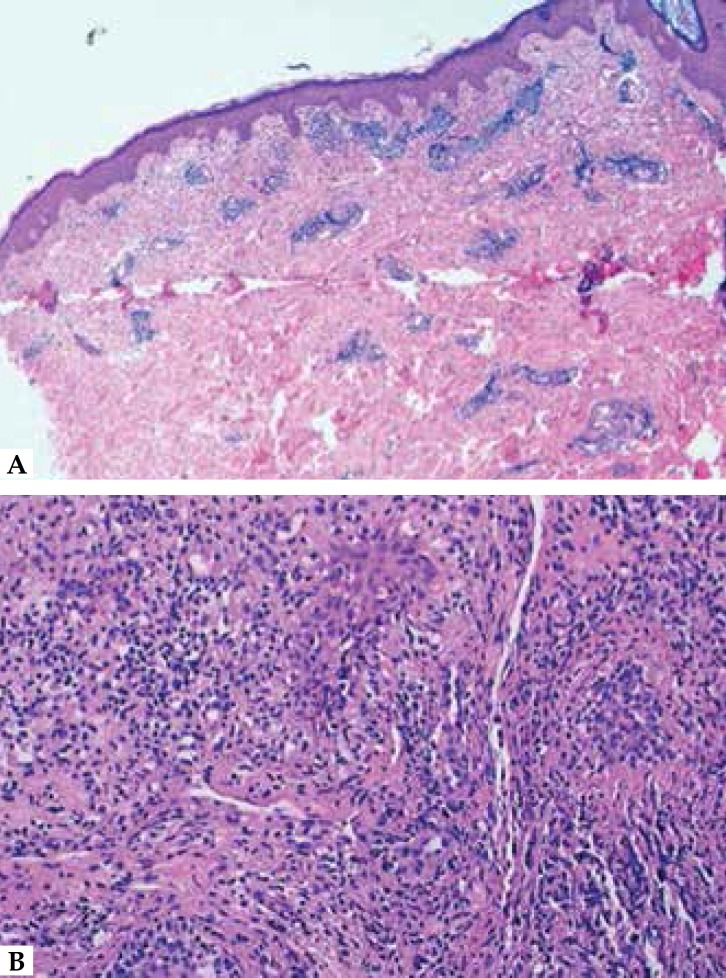
**A.** Superficial and deep dermal infiltrate (x40, H&E); **B.** Monomorphic perivascular and nodular atypical lymphocytic infiltrate (x200, H&E)

**Figure 4:**
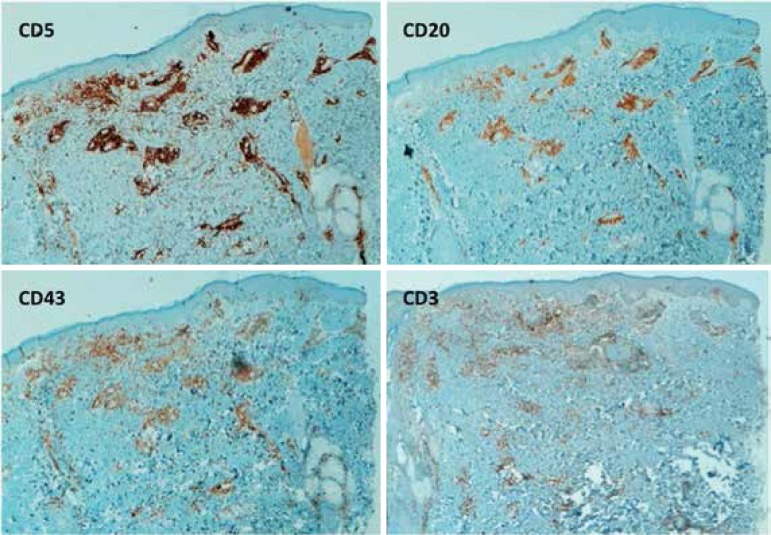
Positive immunological staining for CD5, CD20 and CD43 negative for CD3 (Hematoxylin - eosin, x40)

A few days after the herpetic lesions had healed, the patient developed a monomorphic erythemato-violaceous papular eruption on the scars of the previous herpetic lesions (Figure 2). Histopathological findings and immunohistochemistry studies confirmed B-cell leukemic infiltration (Figures 3 and 4). The lesions cleared under high-dose systemic corticotherapy and the patient remained stable without cutaneous recurrence or evidence of blastic transformation at a 24-month follow-up.

## DISCUSSION

The skin is an active immune organ. Several immune-mediated reactions following different stimuli have been reported, such as the well-known Koebner isomorphic response.^2^ On the other hand, Wolf's isotopic response designates the appearance of two subsequent unrelated dermatoses in the same anatomic location. Although the mechanism behind the second dermatosis is unknown, it has been suggested that localized immunologic imbalance following a certain stimulus may cause the disease. Cutaneous leukemic infiltration after a viral infection has been attributed to recruitment of malignant B-lymphocytes to the skin in response to viral antigenic stimuli.^4^ Other authors discussed the hypothesis of neural damage induced by herpes virus, which would cause local immunosuppression and lead to neoplastic infiltration.^5^

Some cases of B-cell leukemic infiltrates following herpetic infection have been published in the last two decades. Information on follow-up and prognosis was reported for eight patients, of which six experienced resolution of cutaneous lesions, and the clinical prognosis did not appear to worsen.^4,6,7^ On the other hand, two patients had a poor outcome: one of them died of B-CLL after 24 months^4^ and the other patient – whose disease had been in remission for 3 years before the varicella episode – experienced medullar involvement with poor response to combined chemotherapy, requiring allogenic bone marrow transplant.^5^

In our case, an already existing neoplastic proliferation arose *de novo* on the skin after a herpetic infection. The reported cutaneous leukemic infiltration had no effect on the disease course during follow-up time. Our results corroborate the previous observations of a better prognosis in patients with B-cell cutaneous leukemic infiltration following a herpetic infection perhaps because they reflect a cutaneous reactivity pattern to a stimulus rather than a true metastatic process.^4,7^ These observations should be kept in mind when selecting therapeutic approach in such cases.
